# Statin-Associated Myositis in a High-Risk Cardiovascular Patient: Challenges in Reintroducing Therapy

**DOI:** 10.7759/cureus.86246

**Published:** 2025-06-17

**Authors:** Guanming Qi, Aaida Rao, Sara Tariq, Lizeth Diaz Ledesma

**Affiliations:** 1 Internal Medicine, Guthrie Lourdes Hospital, Binghamton, USA

**Keywords:** hyperlipidemia, hyperlipidemia treatment, peripheral arterial diseases, secondary cardiovascular prevention, statin-induced myositis, statin-induced rhabdomyolysis

## Abstract

Statin-associated muscle symptoms (SAMS) are a common side effect of statin therapy, which is widely used to manage cardiovascular diseases (CVDs). These symptoms, which can range from mild muscle discomfort to more severe complications, may lead some patients to discontinue statin therapy, potentially affecting cardiovascular risk management. We present the case of a 67-year-old male patient with multiple cardiovascular comorbidities, including peripheral artery disease (PAD), who developed SAMS after resuming rosuvastatin. Management included statin discontinuation, supportive care with intravenous fluids, and transition to a non-statin lipid-lowering agent (proprotein convertase subtilisin/kexin type 9 (PCSK9) inhibitor). This case highlights the diagnostic and therapeutic challenges of managing SAMS in a patient with complex cardiovascular comorbidities, particularly focusing on distinguishing myopathy from ischemic symptoms and navigating statin reintroduction versus alternative lipid-lowering strategies.

## Introduction

Statin therapy has become the cornerstone for managing hyperlipidemia and reducing cardiovascular risk, especially in patients with established cardiovascular disease (CVD) or peripheral artery disease (PAD). Statins effectively lower low-density lipoprotein cholesterol (LDL-C), improve endothelial function, and stabilize atherosclerotic plaques, significantly reducing morbidity and mortality. However, a common challenge in statin therapy is statin-associated muscle symptoms (SAMS), a clinical diagnosis often based on subjective symptom reporting. While muscle complaints are frequent, true statin-induced myopathy, with objective evidence such as elevated creatine kinase (CK), is rare. SAMS represents a spectrum of muscle-related conditions, ranging from myalgia (muscle discomfort without CK elevation) to myopathy (muscle symptoms accompanied by a mild to moderate CK elevation, typically less than 10 times the upper limit of normal) and rhabdomyolysis, the most severe form, characterized by marked CK elevations (often exceeding 10,000 U/L) and potential for acute renal injury. 

The incidence of SAMS varies, with observational studies estimating muscle-related symptoms in 10% to 20% of patients, while controlled trials suggest true pharmacologic SAMS occurs in only 1% to 2% [1,2]. Risk factors include higher statin doses, older age, female sex, renal dysfunction, and interactions with medications or supplements that impair statin metabolism, for instance, inhibitors of cytochrome P450 3A4 (CYP3A4) or cytochrome P450 2C9 (CYP2C9), as well as substrates of organic anion transporting polypeptides (OATPs), such as OATP1B1. Reintroduction after SAMS is challenging and may involve using alternate statins, intermittent dosing, correcting vitamin D deficiency, or transitioning to non-statin agents like ezetimibe or proprotein convertase subtilisin/kexin type 9 (PCSK9) inhibitors. This case report discusses these strategies in the context of a 67-year-old man with high atherosclerotic CVD (ASCVD) risk and recurrent SAMS. 

## Case presentation

**Figure 1:**
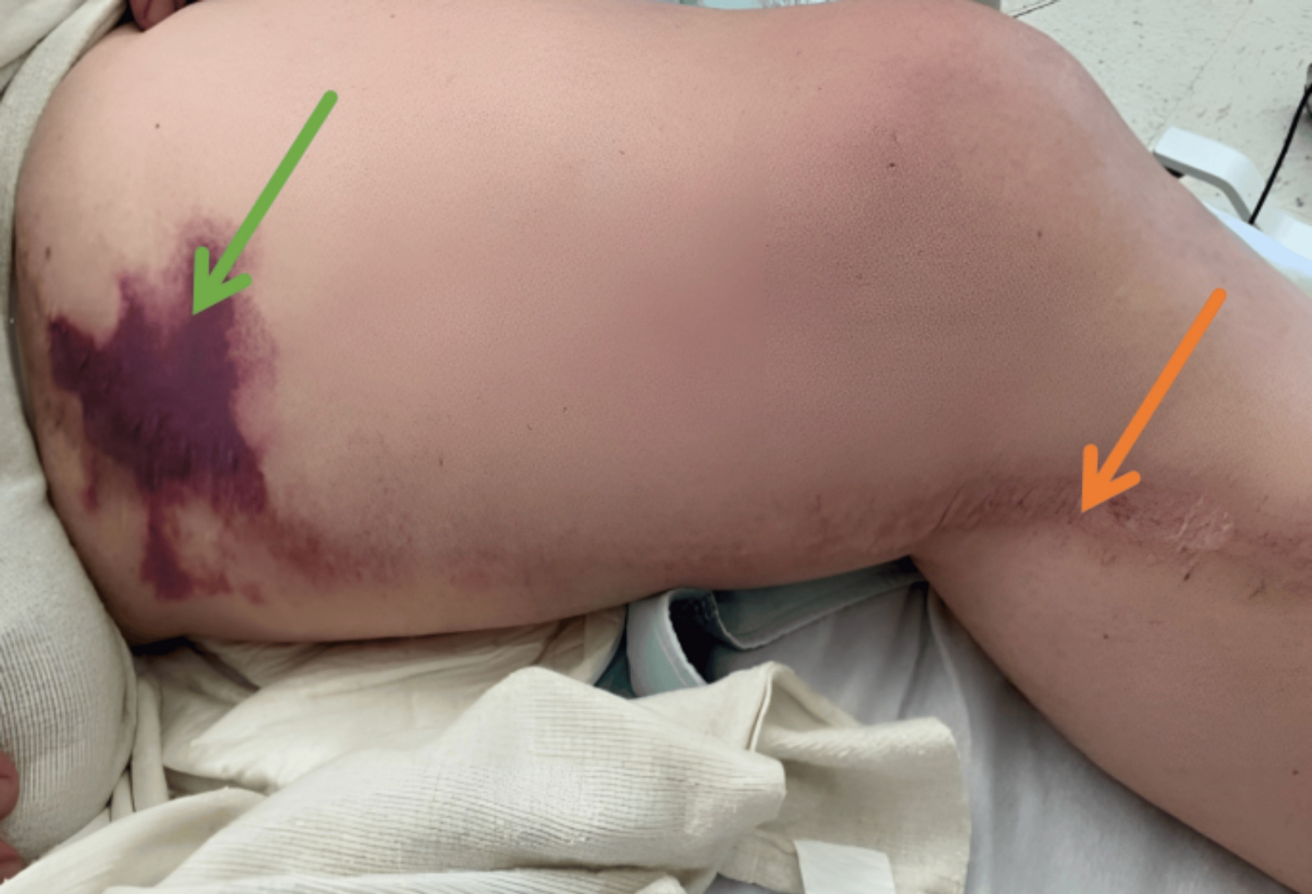
Ecchymosis on the left medial thigh in a patient with peripheral vascular disease and prior statin use (green arrow), along with a healed surgical scar from a previous left femoral-posterior tibial bypass graft and popliteal aneurysm ligation (orange arrow).

A 67-year-old male patient with a history of hypertension, hypertriglyceridemia, obesity, prior non-ST-elevation myocardial infarction (status post coronary artery bypass grafting three years ago), PAD (status post left femoral-posterior tibial bypass and popliteal aneurysm ligation two years ago), and lymphedema presented to the emergency department on May 22, 2025, with sudden-onset, severe left thigh pain. The pain began the previous day while he was working on his car, while wearing a compression wrap for lymphedema. He described the pain as stabbing and accompanied by nausea, but did not complain of any fever or dark urine. His social history included a 25-pack-year smoking history (quit 15 years ago) and chronic alcohol use (three to four beers daily). Long-term medications included aspirin, rosuvastatin, and fenofibrate. Rosuvastatin had been discontinued four months earlier due to self-reported cognitive symptoms, which had been resolved by this admission. Physical examination showed an ecchymosis near the groin on the left medial thigh, diffuse left thigh tenderness, and intact bilateral distal pulses (Figure 1).

**Figure 2:**
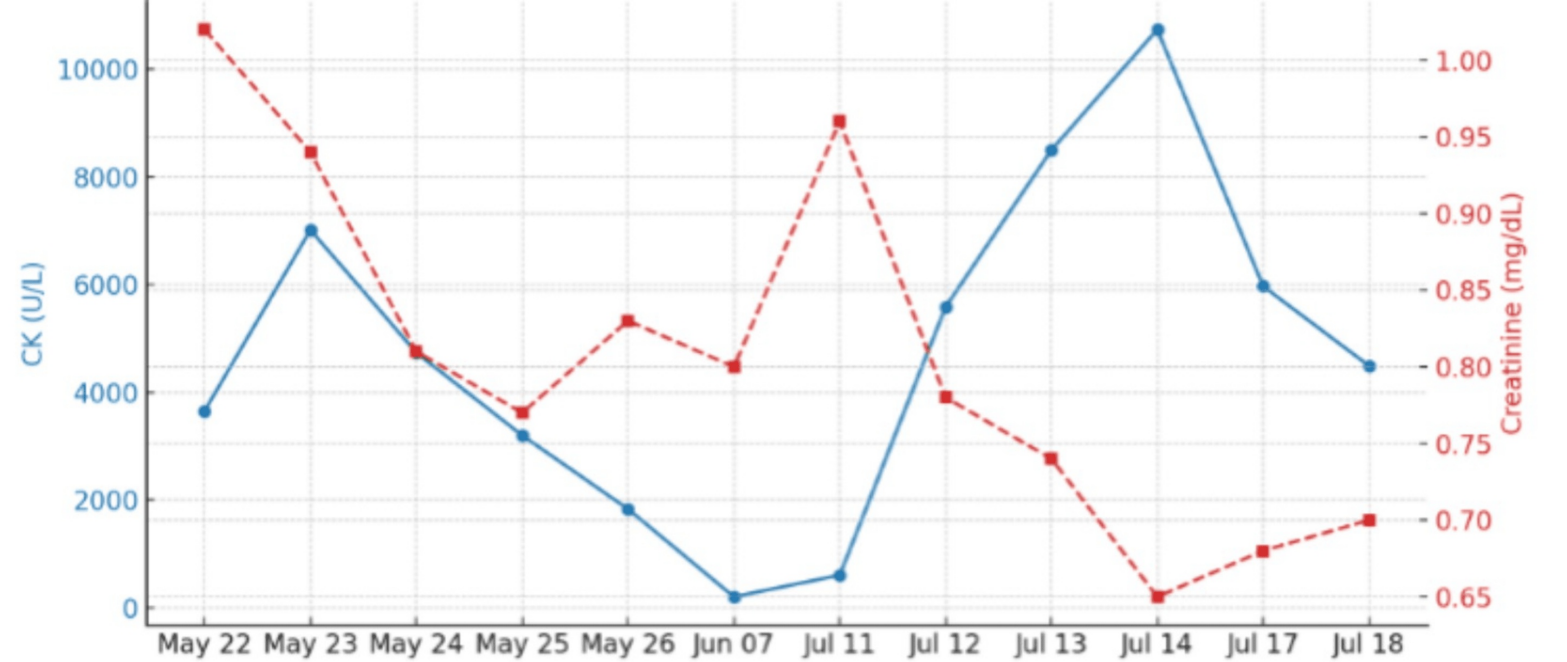
The graph illustrates the trend of creatine kinase (CK) levels (blue line, left y-axis) and creatinine levels (red dashed line, right y-axis) over time. CK levels initially declined to the normal range after discontinuation of statin therapy but surged again following statin reintroduction. Meanwhile, creatinine levels remained relatively stable throughout the observation period.

**Figure 3:**
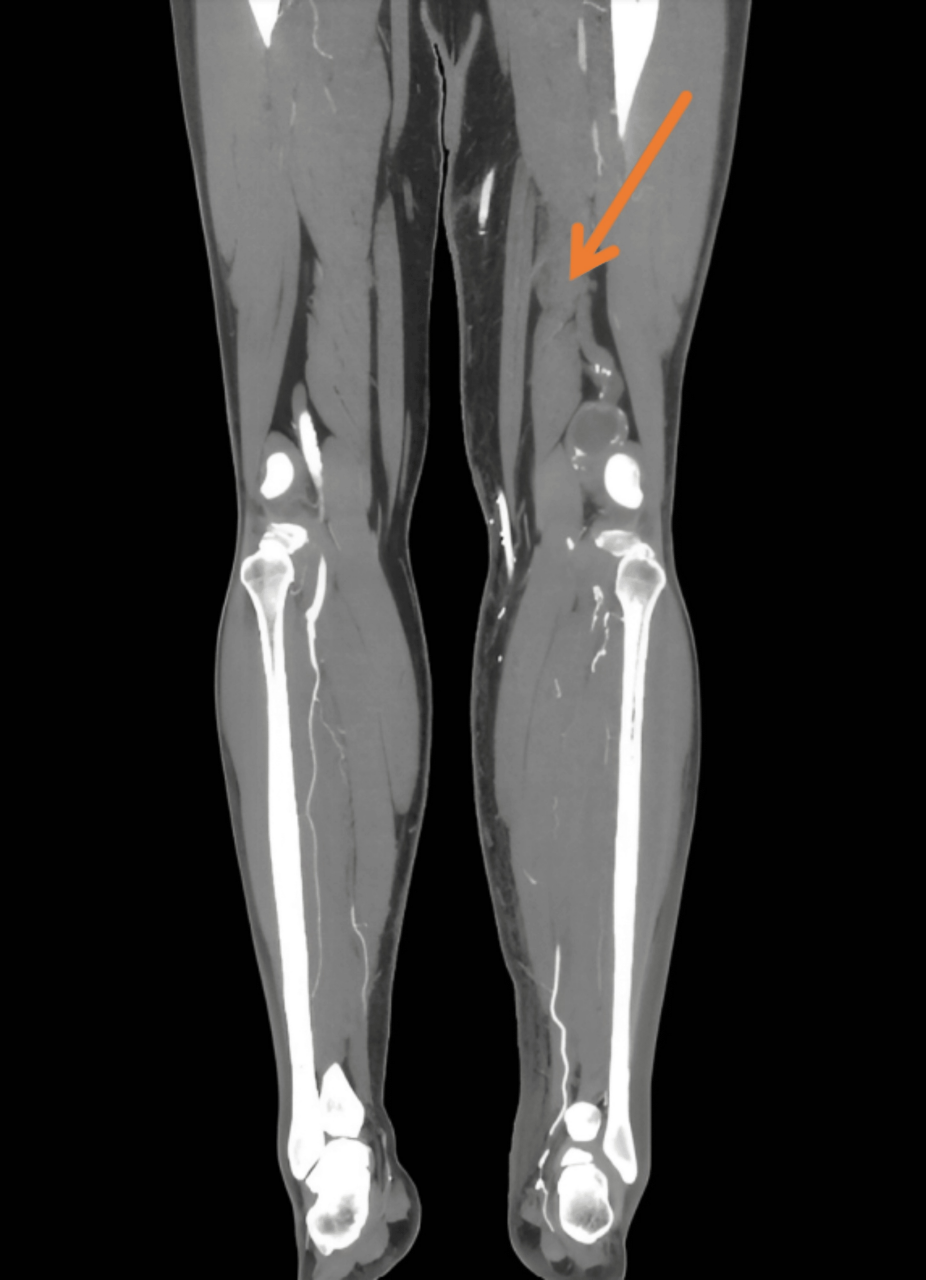
Computed tomography angiogram (coronal view) of the lower extremities showing some irregularity in the muscular contours of the sartorius and gracilis muscles (orange arrow).

There were no focal neurological deficits, such as decreased or exaggerated reflexes, loss of sensation, or muscle atrophy, or no signs of compartment syndrome, such as tense swelling, pain out of proportion, or pain with passive stretch, which reduced concern for acute surgical emergencies. Laboratory findings revealed leukocytosis and elevated CK levels, with preserved renal function (Table 1, Table 2, Figure 2). Infectious etiologies such as cellulitis and infectious myositis were considered; however, the absence of fever, localized erythema, fluctuance, or systemic signs of sepsis, along with unremarkable soft tissue imaging, made infection less likely. Lower extremity venous ultrasound ruled out deep vein thrombosis (DVT), and non-vascular ultrasound showed no hematoma. CT angiography of the lower extremities demonstrated a patent bypass graft, a chronically thrombosed popliteal aneurysm, occlusion of the mid/distal superficial femoral artery, and severe multifocal tibial artery stenosis, findings consistent with known chronic peripheral artery disease rather than acute vasculopathy (Figure 3). These findings, along with preserved distal pulses and the absence of rest pain or skin changes, made acute vascular insufficiency less likely as the primary cause of the patient's thigh pain. Although the onset of symptoms initially coincided with compression wrap use, imaging did not suggest focal ischemia or compartment syndrome. Based on his clinical signs and symptoms and elevated CK level of 2000-3000 U/L, a diagnosis of rhabdomyolysis was made. However, whether it was traumatic rhabdomyolysis or due to concomitant fibrate and alcohol use was still debatable. He was treated conservatively with intravenous fluids and analgesia, and fenofibrate was held. CK levels gradually declined over five days.

**Table 1:** Complete blood count (CBC) on the admission day WBC: white blood cell; RBC: red blood cell; HGB: hemoglobin; HCT: hematocrit; MCV: mean corpuscular volume; MCHC: mean corpuscular hemoglobin concentration; PLT: platelets; NEUT: neutrophils; LYMPH: lymphocytes; mono: monocytes; EO: eosinophils; BASO: basophils; μl: microliter; g/dl: grams per deciliter; FL: femtoliter; %: percentage

CBC with differential	Results	Reference range
WBC (10^3^/μL)	13.32	4.0-11.0
RBC (10^6^/μL)	5.37	4.7-6.1 (men), 4.2-5.4 (women)
HGB (g/dL)	15.6	13.8-17.2 (men), 12.1-15.1 (women)
HCT (%)	46.8	40-54 (men), 36-48 (women)
MCV (fL)	87.2	80-100
MCHC (g/dL)	33.3	32-36
PLT (10^3^/μL)	201	150-450
NEUT (%)	80.2	40-70
LYMPH (%)	10.8	20-40
MONO (%)	5.3	2-8
EO (%)	1.4	1-4
BASO (%)	0.6	0.5-1

**Table 2:** Comprehensive metabolic panel (CMP) on the admission day mmol/L: millimoles per liter; mg/dL: milligrams per deciliter; U/L: units per liter; g/dL: grams per deciliter.

Comprehensive metabolic profile	Results	Reference range
Sodium (mmol/L)	141	136-145
Potassium (mmol/L)	3.7	3.5-5.1
Calcium (mg/dL)	10.0	8.4-10.4
Chloride (mmol/L)	106	98-110
Carbon dioxide (mmol/L)	22	20-30
Glucose (mg/dL)	144	70-99
Creatinine (mg/dL)	1.02	0.72-1.25
Urea (mg/dL)	13.71	8-26
Alanine aminotransaminase (U/L)	21	11-55
Aspartate aminotransferase (U/L)	32	5-34
Alkaline phosphatase (U/L)	66	40-150
Total bilirubin (mg/dL)	0.5	0.2-1.2
Total protein (g/dL)	7.2	6-8

At a follow-up visit, given his high cardiovascular risk profile and previously resolved cognitive symptoms, his primary care physician and cardiologist jointly decided to reinitiate rosuvastatin at a lower dose and fenofibrate. The suspicion for statin-induced rhabdomyolysis was low, as the patient was not on statins four months prior to the rhabdomyolysis. The decision to benefit from lipid-lowering therapy outweighs the risks, as the patient had a positive family history, a coronary bypass graft, and PAD. However, the decision aimed to achieve aggressive lipid-lowering therapy, although the simultaneous reintroduction of both agents represented a high-risk strategy. The patient remained symptom-free for approximately six weeks. On July 11, he was readmitted with recurrent left leg pain. Within two days, CK levels exceeded 10,000 U/L, consistent with rhabdomyolysis; however, renal function remained stable, suggesting that early and aggressive intravenous fluid management was effective in preventing acute kidney injury (Figure 2). MRI of the left thigh revealed extensive edema in the quadriceps, sartorius, and gracilis muscles, without tendon rupture or abscess, findings radiologically suggestive of myositis. However, given the degree of CK elevation, a diagnosis of rhabdomyolysis, likely multifactorial in origin, was made. Contributing factors may have included statin re-exposure, fenofibrate coadministration, and chronic alcohol use, all of which can predispose to muscle injury. He was managed with intravenous fluids, discontinuation of rosuvastatin, and counseling for alcohol cessation. Although we recognize this may have carried additional myotoxic risk, fenofibrate was resumed to manage his persistent hypertriglyceridemia at levels greater than 300, which could put him at risk of pancreatitis if not lowered. CK levels declined below 5,000 U/L, and he was discharged after one week with referrals to physical therapy and pain management. Given the recurrence of statin-associated muscle injury, a PCSK9 inhibitor (alirocumab) was recommended as an alternative lipid-lowering therapy.

## Discussion

This case illustrates the complexity of managing SAMS in a high-risk cardiovascular patient with PAD, prior vascular reconstruction, persistently high triglycerides, and chronic alcohol use. The patient experienced recurrent SAMS episodes, initially with rhabdomyolysis with CK levels of 2,300 U/L and subsequently exceeding 10,000 U/L and MRI findings of myositis, indicative of rhabdomyolysis with myositis, following reinitiation of both rosuvastatin and fenofibrate. Despite adequate hydration and preserved renal function, this clinical course underscores the importance of careful medication reintroduction strategies and awareness of potential drug interactions in patients with prior SAMS. The combination of rosuvastatin and fenofibrate likely contributed to the recurrence of rhabdomyolysis in addition to myositis, as both agents possess independent myotoxic potential and share metabolic pathways such as glucuronidation, thereby amplifying toxicity risk [1,2]. Additionally, the patient’s chronic alcohol use may have predisposed him to muscle injury via mitochondrial dysfunction [2]. MRI findings of diffuse, non-enhancing thigh muscle edema supported a diagnosis of toxic or metabolic myopathy rather than ischemic or infectious causes. 

Clinical guidelines strongly recommend statin therapy for secondary prevention in individuals with a history of myocardial infarction, stroke, or other atherosclerotic cardiovascular conditions. However, despite high risk for ASCVD events, patients with PAD are less likely to be prescribed statins compared to those with coronary heart disease (CHD) or cerebrovascular disease [3]. This discrepancy may reflect clinical hesitancy stemming from concerns about statin-induced muscle toxicity. Our patient had PAD along with CAD, which warranted statin use. Nonetheless, the benefits of statins in patients with advanced PAD, including improvement in graft patency following infrainguinal bypass with saphenous vein, are well established in observational studies and supported by vascular surgery guidelines [4]. Consequently, despite the risk of muscle-related side effects, continuing statin therapy is generally recommended to optimize vascular outcomes and reduce cardiovascular morbidity [4]. Distinguishing SAMS from PAD-related ischemic symptoms is clinically important but challenging. Ischemic claudication typically manifests as exertional calf or thigh pain that resolves with rest. In contrast, SAMS may occur at rest, involve multiple or unusual muscle groups, and present with diffuse tenderness or proximal weakness, key features that aid in the differential diagnosis [5]. 

In this case, the patient was restarted on high-intensity rosuvastatin without interim CK monitoring or a stepwise titration approach, which represented a high-risk strategy. Current guidelines recommend that patients with a history of SAMS be re-challenged cautiously, ideally using lower-intensity statins (e.g., pravastatin, fluvastatin), alternate-day dosing, or switching to statins with lower lipophilicity and less reliance on CYP450 metabolism, all of which may lower the risk of recurrent symptoms [6-8]. CK levels should be rechecked two to four weeks after reintroduction [6], with ongoing assessment of muscle symptoms and renal function. Some evidence supports the use of long-acting statins (e.g., rosuvastatin or fluvastatin) on an intermittent schedule, and correcting contributing factors such as vitamin D deficiency may improve tolerability [9]. Importantly, CK levels do not always correlate with symptom severity, complicating monitoring. Although the patient initially tolerated rosuvastatin, symptoms returned within six weeks and were more severe, leading to hospitalization. While fenofibrate was continued for hypertriglyceridemia of greater than 300 U/L, this decision must be balanced against its additive myotoxic risk. 

**Figure 4:**
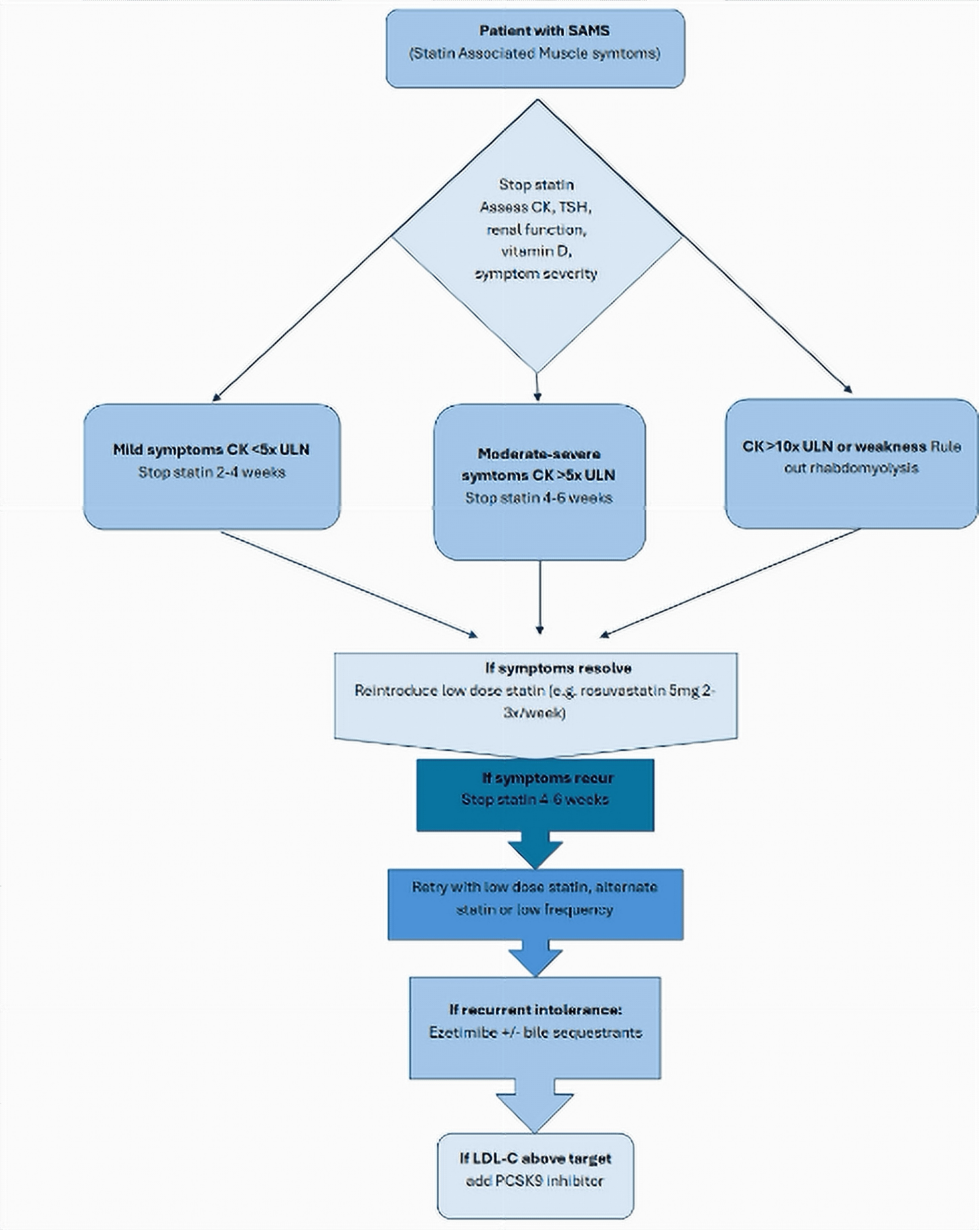
Flowchart showing management of patient with statin-associated muscle symptoms (SAMS) CK: creatinine kinase; TSH: thyroid stimulating hormone; ULN: upper limit of normal; LDL-C: low density lipoprotein cholesterol; PCSK9: proprotein convertase subtilisin/ kexin type 9 This algorithm has been created by the authors with content adapted from [12-15] in addition to the current guidelines.

In patients with confirmed statin intolerance, PCSK9 inhibitors provide an effective alternative. In this case, alirocumab was recommended. These agents have demonstrated significant reductions in cardiovascular events in high-risk populations, as highlighted in the FOURIER trial [10]. However, real-world barriers such as cost, insurance authorization, and injection adherence must be considered [11]. A generalizable approach to managing SAMS in high-risk patients includes ruling out secondary causes (e.g., hypothyroidism, vitamin D deficiency, drug interactions); statin discontinuation followed by symptom monitoring; CK, renal function, and symptom surveillance during rechallenge; stepwise reinitiation using low-intensity or intermittent statin dosing; and transition to non-statin agents (e.g., ezetimibe, PCSK9 inhibitors) if intolerance persists (Figure 4) [12-15]. 

## Conclusions

This case highlights the challenge of managing SAMS in a patient with PAD, prior vascular surgery, and recurrent rhabdomyolysis. The decision to resume high-potency statin therapy despite prior SAMS led to recurrence, illustrating the need for individualized risk-benefit assessment. While statins remain central to cardiovascular prevention, safer reintroduction strategies and monitoring protocols are essential. PCSK9 inhibitors were ultimately selected as a more tolerable alternative with demonstrated cardiovascular benefit. Clinicians should avoid high-risk drug combinations (e.g., statin-fibrate) in SAMS-prone patients and ensure regular monitoring of CK, renal function, and muscle symptoms within two to four weeks of reinitiation. This case reinforces the need for shared decision-making and careful therapeutic selection in patients with complex cardiovascular risk profiles and previous statin intolerance. 
